# Mental health night: A peer-led initiative

**DOI:** 10.36834/cmej.70208

**Published:** 2020-12-07

**Authors:** Hannah Kearney, Becky Jones, Jia Hong Dai, Ivana Burcul

**Affiliations:** 1 Michael G. DeGroote School of Medicine, Niagara Regional Campus, Ontario, Canada

## Implication Statement

We describe a peer-led mental health (MH) workshop that was held at the Michael G. DeGroote School of Medicine (Niagara Regional Campus) in collaboration with Student Affairs. Workshop aims included facilitating discussions among peers and engaging in case-based learning about MH experiences in medical school. Post-workshop, attendees reported increased comfort in talking to classmates about personal MH, recognizing MH crises, and asking for help from peers. We believe that engaging medical learners in MH discussions early on in medical education is critical, and that peer-led workshops may aid in decreasing future MH difficulties and burnout. Due to the low-cost of holding peer-led workshops, this event could be easily replicated at other training sites.

## Énoncé des implications de la recherche

Nous décrivons un atelier sur la santé mentale dirigé par des pairs qui s’est tenu à l’école de médecine Michael G. DeGroote (Campus régional de Niagara) en collaboration avec le service des affaires étudiantes. Les objectifs de l’atelier étaient de faciliter les discussions entre pairs et de d’encourager l’engagement dans une expérience d’apprentissage par discussion de cas utilisant l’apprentissage expérientiel en santé mentale durant le cours de médecine. À l’issue de l’atelier, les participants ont déclaré se sentir plus à l’aise pour parler de leur santé mentale avec leurs pairs, reconnaître les crises de santé mentale et demander l’aide de leurs pairs. On croit qu’il est essentiel que les étudiants en médecine prennent part aux discussions sur la santé mentale dès le début du programme d’études en médecine et que les ateliers dirigés par des pairs peuvent contribuer à réduire leurs potentiels problèmes de santé mentale et d’épuisement. En raison du faible coût des ateliers dirigés par des pairs, cet événement pourrait facilement être reproduit dans d’autres sites de formation.

## Introduction

Researchers have reported that medical students (MS) are a high-risk group for mental health (MH) challenges. Compared to other postsecondary graduates, MS experience higher levels of psychological distress, mood/anxiety disorders, and suicidal ideation.^1^ In Canada, 39.4.% of first-year students disclosed some degree of psychological distress.^1^ Moreover, the prevalence of burnout during medical school has been reported as high as 44.2%.^2^ Promoting prevention, recognizing symptoms, and destigmatizing psychological distress are crucial actions that encourage MS to seek help.^2^ We investigated the effectiveness of a peer-led workshop on improving first-year MS’ comfort discussing MH.

## Methods

We held an optional, two-hour, peer-led MH workshop on-campus for all 28 first-year MS at Michael G. DeGroote School of Medicine’s Niagara Regional Campus (NRC), halfway through the year. Students were in pre-clerkship, which consists of lectures and small group learning (no designated clinical time). Four second-year NRC students hosted the workshop, presented an introduction to MH in medical school, and shared personal narratives. The hosts also developed case scenarios about coping strategies and recognizing peers in need, based on personal experiences and situations MS may encounter during their training. The hosts facilitated small group discussions about the scenarios. Due to the sensitive nature of the workshop, the NRC Student Affairs Director was present during the workshop and accessible via email post-workshop for student support.

Pre-workshop, attendees completed the Medical Student Stressor Questionnaire (MSSQ)^3^, a validated 40-item self-report survey designed to identify sources of stress in MS across six domains (Academic Related, Interpersonal & Intrapersonal Related, Teaching and Learning Related, Social Related, Drive & Desire Related, and Group Activities Related Stressors). Additionally, students completed a novel wellness questionnaire (NWQ) pre- and post-workshop. Created by the workshop hosts, the NWQ was designed to evaluate the efficacy of the workshop. Because the NWQ has not been validated, a retrospective face validity assessment regarding the clarity of the NWQ was performed via anonymous peer-review by eight second-year MS and was deemed to be clear and understandable. This project was identified as quality improvement by the Hamilton Integrated Research Ethics Board, and was thus exempt from ethics review.

## Results

Twenty students attended the workshop. Fifteen students completed the MSSQ, and fourteen students completed the NWQ. On the MSSQ, students indicated higher stress levels in the academic and teaching/learning domains, compared to the intrapersonal/interpersonal, social, drive/desire, and group-activity domains. The workshop significantly impacted (*p* < 0.05) students’ comfort levels talking to classmates about personal MH, recognizing MH crises, and asking for help from friends for personal MH (Table 1).

**Table 1:** Wilcoxon signed-rank tests of the novel Wellness Questionnaire pre- and post-session

Questions	Median, Pre-session	Median, Post-session	Asym. Sig (2 tailed)
I feel comfortable talking about my own mental health in general.	5.0	5.0	0.163
I feel comfortable talking about my own mental health with my classmates.	4.5	5.0	0.041*
I feel comfortable talking to others about their mental health.	6.0	6.0	0.577
I feel comfortable talking to my classmates about their mental health.	6.0	6.0	1
I feel comfortable asking for help from my friends for my own mental health problems.	4.5	5.0	0.035*
I feel comfortable seeking professional help for my own mental health problems.	5.0	5.0	0.595
I am comfortable recognizing mental health crises.	4.5	5.0	0.033*
I feel comfortable expressing my concern to a friend about their mental health.	5.0	5.0	0.023*
I feel comfortable talking to patients about mental health.	5.0	5.5	0.157
I feel comfortable helping a friend with a mental health problem.	5.0	6.0	0.262
I feel like I know a lot about mental health.	4.5	4.0	0.206
I recognize when I’m having a bad mental health day.	5.0	6.0	0.063
I use strategies to help me with my mental health.	6.0	6.0	0.589
I feel more comfortable talking about mental health since I started medical school.	4.0	4.0	0.024*

(n = 14). Statistical significance denoted by bolded text and asterisk (p < 0.05).

## Discussion

This study demonstrates that a peer-led mental health workshop for pre-clerks can increase MS comfort in talking to classmates about personal MH, in recognizing MH crises, and in asking for help from peers. These findings support previous work that has identified peer-led wellness initiatives as a potential method for addressing MS well-being.^4^ Our intervention improved students’ comfort in discussing MH with peers; however, some students reported continued discomfort helping peers with MH and seeking professional care. This may be due to the limited discussion of these topics during the workshop. We suggest that facilitators of future workshops address helping peers with MH during the case-based learning session, and how to seek professional care by providing students with actionable steps to accessing local MH care.

Although our study was limited by a small voluntary response sample and use of the NWQ, we believe other institutions could benefit from hosting similar workshops. Having upper-year MS host the workshop can help foster a collegial atmosphere, and we believe that using voluntary attendance provides students with a unique learning opportunity (versus an additional academic burden). In the future, we hope to evaluate NWQ validity and to assess for longitudinal effects of the workshop.

## References

[1] Maser B, Danilewitz M, Guérin E, Findlay L, Frank E Medical student psychological distress and mental illness relative to the general population: a Canadian cross-sectional survey. Acad Med. 2019 11 1;94(11):1781-91. 10.1097/acm.0000000000002958. 31436626

[2] Frajerman A, Morvan Y, Krebs MO, Gorwood P, Chaumette B Burnout in medical students before residency: a systematic review and meta-analysis. Euro Psych. 2019 1;55:36-42. 10.1016/j.eurpsy.2018.08.006 30384110

[3] Yusoff MS, Rahim AF, Yaacob MJ The development and validity of the medical student stressor Questionnaire (MSSQ). ASEAN Journal of Psychiatry. 2010 1 1;11(1):231-5. https://pdfs.semanticscholar.org/4c7e/3c6a2948bd424adec12e067eb1b9cd4d8f58.pdf [Accessed on April 5 2020].

[4] Wiederhold BK, Cipresso P, Pizzioli D, Wiederhold M, Riva G Intervention for physician burnout: A systematic review. Open Med. 2018 7 4;13(1):253-63. 10.1515/med-2018-0039 PMC6034099 29992189

